# Jujube Syrup and Starter YF‐L922 Co‐Fermentation of Yak Yogurt: Effects of Quality Properties, Antioxidative Activities and Structure

**DOI:** 10.1002/fsn3.4589

**Published:** 2024-11-08

**Authors:** Xiaolin Liang, Bo Ding, Songxuan Li, Hao Zhang, Jialin Bai, Jutian Yang, Dandan Gao, Jiajia Song, Hongna Liu

**Affiliations:** ^1^ Key Laboratory of Biotechnology and Bioengineering of State Ethnic Affairs Commission, Biomedical Research Center Northwest Minzu University Lanzhou China; ^2^ China‐Malaysia National Joint Laboratory, Biomedical Research Center Northwest Minzu University Lanzhou China; ^3^ College of Life Science and Engineering Northwest Minzu University Lanzhou Gansu China; ^4^ College of Food Science and Nutritional Engineering China Agricultural University Beijing China; ^5^ Engineering Research Center for key Technology and Industrialization of Cell‐Based Vaccine Northwest Minzu University, Ministry of Education China; ^6^ College of Food Science Southwest University Chongqing China

**Keywords:** bioactive properties, fermented yak milk, jujube syrup, physicochemical properties, sensory science

## Abstract

Different percentages of jujube yrup (0%, 3%, 6% and 9%) were incorporated into yak milk and fermented using the fermenting agent **YF‐L922**. The quality characteristics and antioxidant activity of the resulting yogurt were evaluated at days 0, 7, 14, 21 and 28. The results indicated that the pH and acidity of the yogurt were not significantly influenced by the varying additions of jujube syrup during storage (*p* > 0.05). However, the addition of jujube syrup significantly reduced the water‐holding capacity of the yogurt (*p* < 0.05). Furthermore, the levels of jujube syrup were significantly and positively correlated with both antioxidant activity and free radical scavenging ability (*p* < 0.05). The live bacterial count of the yogurt decreased significantly by day 28, although the count of live lactic acid bacteria remained above 10^6^ CFU/mL. Notably, yak yogurt with a 3% addition of jujube syrup achieved a favorable sensory score. The incorporation of jujube syrup resulted in a firmer texture and a more porous microstructure, demonstrating a higher degree of syneresis. Additionally, the inclusion of jujube syrup substantially diminished the animalic odor associated with yak milk, improved flavor acceptability and enhanced the antioxidative properties of yak yogurt. Therefore, yak yogurt augmented with jujube syrup represents a novel product with high nutritional value.

## Graphical abstract

Different percentages of jujube syrup (0%, 3%, 6% and 9%) were added to yak milk and fermented with the fermenting agent (YF‐L922) and the quality characteristics and antioxidant activity were determined for yogurt at days 0, 7, 14, 21 and 28 days. The mixing of jujube syrup reduced the animalic (yak)‐odor of yak milk considerably, improved the flavor acceptability and enhanced the antioxidative properties of yak yogurt. Thus, yak yogurt combined with jujube syrup addition represents a new yak milk product with high value and high nutrition.

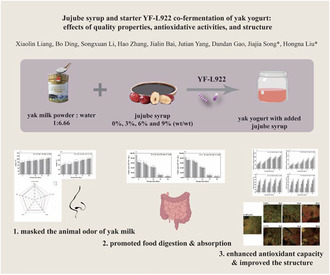

## 1 Introduction

Yak (*Bos grunniens*) is the dominant species in the alpine pasture ecosystem of the Tibetan Plateau in China (Tian et al. 2023). It is estimated that approximately 90% of the world's total population, exceeding 15 million yaks, is found in China, primarily in the northwest region (Fan et al. 2020). Yak milk and its products are essential sources of daily nutrients for the survival of residents. Compared to cow milk, yak milk offers higher nutritional value than Holstein milk, containing 1.5 times more total casein, 2 times more average fat and 6 times more Fe (Li, Zong et al. 2023). However, yak milk is characterized by low yield and challenges in storage and maintaining freshness. Consequently, the deep processing of yak milk to develop high‐value‐added products has become a pressing need. Most fresh milk is typically processed into storable products such as milk powder, ghee and Qula (Guo et al. 2014). However, yak milk possesses an undesirable flavor due to the presence of short‐chain fatty acids, which limits its acceptance among consumers and hinders the development of yak dairy products (Liu et al. 2011).

With the increase in living standards, people's dietary preferences are gradually shifting toward natural, healthy and nutritious foods, leading to the growing popularity of probiotic products. Yogurt, with its unique flavor and nutrient‐rich profile, enhances gastrointestinal motility, promotes food digestion, absorption and improves lactose intolerance is widely preferred by consumers (Kaur et al. 2020). The YF‐L922 culture consists of *Lactobacillus bulgaricus* and *Streptococcus thermophilus* in a 1:1 ratio, both of which are widely recognized as important fermenting strains in dairy production. They possess significant economic and research value as probiotics within the lactic acid bacteria (LAB) and as key microorganisms in the human intestine. Notably, these bacteria engage in a reciprocal symbiotic relationship: *L. bulgaricus* produces proteolytic enzymes during fermentation, releasing amino acids from casein, which promote the growth of *S. thermophilus*. Concurrently, formate and carbon dioxide produced by *S. thermophilus* enhance acid production in *L. bulgaricus*, ultimately contributing to yogurt's unique flavor and sticky consistency.

Currently, yogurt is often produced with various supplements to enhance flavor and nutritional content, with fruits and herbs being the most common (Ahmad et al. 2022; Kaur et al. 2020). The addition of a mixture of bioactive peptides and lily bulb powder to goat yogurt improves its texture and flavor during storage (Zhao, Cheng et al. 2022). Moreover, dietary fiber incorporated into yogurt can have beneficial effects on human health (Ahmad et al. 2020). Safdari et al. demonstrated that banana fiber and banana peel fiber in camel milk yogurt can increase viscosity, probiotic viability and consistency (Safdari et al. 2021). Yu et al. combined fermented yogurt with peanut sprouts and found that the protein, fat, carbohydrate and calorie content surpassed that of regular yogurt (Yu et al. 2022). Additionally, fruit by‐products have been shown to enhance the structure and reduce syneresis in yogurt (Sah et al. 2016). It has been reported that the addition of apple pomace to stirred yogurt modifies its structure, increases viscosity and reduces whey release during storage (Wang, Kristo, and Lapointe 2020). Overall, the trend of incorporating diverse supplements into yogurt while enhancing its flavor, texture and nutritional value has become a prominent focus of research. Currently, most fruit yogurts available in the market are produced by adding fruits post‐fermentation; however, products **co‐fermented** with fruit and vegetable juices and yak milk are rarely encountered.

Jujube contains substances such as polysaccharides and polyphenols, which are rich in nutritional value and possess strong antioxidant properties (Lu et al. 2021). Furthermore, due to its exceptionally high medicinal value, jujube serves as a functional food that benefits both health and nutrition. Its pleasant aroma is also appreciated by consumers. While jujube is widely grown in China, its limited storage capabilities have led to the emergence of various jujube‐related products, such as jujube syrup (Sobhani et al. 2019), jujube wine (Zhao, Xue et al. 2022) and jujube vinegar (Wang et al. 2022). Additionally, some researchers have discovered that jujube mucilage can function as a natural stabilizer in the production of stirred yogurt (Feng et al. 2019; Yekta and Ansari 2019).

The purpose of this research is to analyze the impact of different concentrations of jujube syrup on the physicochemical properties, bioactive properties and sensory science of yak yogurt during co‐fermentation. The newly developed products not only combine the nutrients of yak milk and jujube but also reduce the unpleasant taste of yak milk while adding the pleasant flavor of jujube. As a result, these products are popular among consumers and present positive business prospects. Furthermore, optimizing the use of jujube and yak milk is of great significance for the advancement of both industries.

## 2 Material and Methods

### 2.1 Raw Materials

The concentrated jujube syrup used in this study was from Stone Floor County Shuter Date Industry Co. Ltd. and yak milk powder was from Gansu Hualing Dairy Co. Ltd. The freeze‐dried starter culture (**YF‐L922**) used in yogurt comprising *Lactobacillus bulgaricus* and *Streptococcus thermophilus*is from Chr. Hansen (Beijing) Trading Co. Ltd.

### 2.2 Preparation of Yak Yogurt Product

According to the ratio of yak milk powder and water (1:6.66), it was brewed and mixed well. Then, jujube syrup concentrate was added to the yak milk at concentrations of 0%, 3%, 6% and 9% (wt/wt). The prepared yak milk was pre‐heated at 65°C for 5 min, homogenized at 25 MPa for 10 min and sterilized at 95°C for 5 min. After sterilization, the sample was cooled to ambient temperature (22°C) to prevent heat from excessive temperature to inactivate bacteria. After that, 0.005 g of YF‐L922 yogurt cultures per 100 g of the cooled sample was inoculated, stirred thoroughly and incubated at 42°C for 6 h to produce the finished jujube syrup yogurt. Yak yogurt was prepared in parallel with three equal portions of each concentration.

### 2.3 Measurement of the Acidity and pH Value

The yak yogurt with jujube syrup in preparation was analyzed for acidity and pH value. The acidity was determined based on Granato and expressed in Thorner degree (°T) (Granato et al. 2018). The pH value was measured using standard procedures. The electrode of the calibrated handheld portable pH meter was inserted directly into the 20 mL yogurt sample. Every parallel measurement was performed three times and averaged.

### 2.4 Microbiological Analysis of Yogurts

Viable counts of LAB were determined on a seven‐day cycle in triplicate. For the LAB counts at each pre‐established time, serial dilutions of each yogurt sample were performed with sterile saline at a ratio of 1:9.

The counts were performed in Modified Chalmers (MC) agar and DeMan, Rogosa and Sharpe (MRS) agar, respectively. *S. thermophilus* colonies were recorded in MC agar for 48 h of culture at 36°C ± 1°C, while *L.bulgaricus* were enumerated on MRS agar after 72 h of culture at 36°C ± 1°C (Bakry, Chen, and Liang 2019). The results were obtained as the number of colony forming units per mL of yogurt (CFU/mL).

### 2.5 Measurement of Syneresis and Water‐Holding Capacity

The measurement of the susceptibility to syneresis was modified according to Isang's method (Isanga and Zhang 2009). 50 g of yogurt was filtered in a funnel and the released whey was measured after 2 h. The susceptibility to syneresis was calculated using the following formula:

$$\text{Susceptibility to syneresis}\left(\%\right)=\frac{\text{weight of the released whey}\left(g\right)}{\text{weight of the yogurt}\left(g\right)}\times 100\%$$

Using the centrifugal approach designed by Moschopoulo to measure water‐holding capacity (Moschopoulou et al. 2018). When the yogurt coagulation was formed, the yogurt at 5000 × *g* was centrifuged for 10 min. Drained away the top of the supernatant and the remainder of the pellets were weighed. The water holding capacity was calculated using the following formula:

$$\text{Water Holding Capacity}\;\left(\%\right)=\frac{\text{weight of the precipitate}\left(g\right)}{\text{weight of the sample}\left(g\right)}\times 100\%$$

### 2.6 Sensory Evaluation

Sensory evaluation of yak yogurt products was conducted throughout the storage period. A tasting panel consisting of 10 trained evaluators was selected to perform a descriptive sensory analysis using quantitative descriptive analysis (QDA). The same team members conducted the sensory assessments under controlled conditions of temperature and lighting to ensure consistency. The evaluation criteria included color, texture, odor and taste, with results expressed as mean scores.

### 2.7 Antioxidant Activity Determination

The yak yogurt combined with jujube syrup was weighed at 2.0 g and mixed with 6 mL of methanol (80%, v/v). Afterward, the samples were ultrasonicated at 50 W for 20 min and centrifuged at 5000 × *g* for 15 min. The supernatant was taken and used to determine the antioxidant activity.

#### 2.7.1 DPPH Free Radical Scavenging Activity Assay

The antioxidant activity of DPPH‐inhibited yak yogurt samples was determined using the method refined from Plank (Plank et al. 2012). Methanolic extract of 0.5 mL prepared from yak yogurt samples was mixed completely with 4.0 mL (0.5 mmol/L) of methanolic solution of DPPH. For the analysis, the mixture was then adjusted to 10.0 mL with distilled water and incubated for 30 min at room temperature and away from light. The absorbance values were measured by spectrophotometer at 517 nm using distilled water as a blank.

The ability to remove DPPH radicals was shown as the percentage suppression of DPPH radicals. The formula for calculating the percentage suppression of DPPH radicals by yak yogurt samples was based on the absorbance values of the yak yogurt samples as follows:

$$\text{DPPH radical scavenging rate}\;\left(\%\right)=\frac{\left(A-\mathrm{As}\right)}{A}\times 100$$

A represents the absorbance of the DPPH solution, As represents the absorbance of the mixed sample solution after 30 min.

#### 2.7.2 Ferric Reducing Antioxidant Power (FRAP) Assay

FRAP determination was performed by the modified method described by Lee (Lee et al. 2021). 0.5 mL of methanolic extract of yak yogurt and 3.0 mL of prepared FRAP reagent (mixed with 0.3 mol/L of acetic acid buffer, 10 mmol/L of TPTZ reagent and 20 mmol/L of FeCl_3_ solution in the ratio of 10:1:1 in volume) were combined and placed in a water bath at 37°C for 10 min. Absorbance was measured at 593 nm, while distilled water was used as blank. Ferric‐reducing antioxidant power was expressed using the concentration of ferric calculated from the standard curve.

#### 2.7.3 Trolox Equivalent Antioxidant Capacity Assay

Experiments were performed according to Sahingil with small modifications (Sahingil and Hayaloglu 2022). The solution of 7 mmol/L ABTS and 2.45 mmol/L potassium persulfate was prepared, mixed in the ratio of 1:2 by volume and placed in a light‐proof environment for 16 h. Diluted with ethanol before use to achieve an absorbance of 0.70 ± 0.02 at 734 nm. Then, 0.6 mL of methanolic extract of yogurt was pipetted and 2.4 mL of diluted ABTS solution was added, which was reacted for 6 min under 22°C. Recorded the number at 734 nm at this time.

The ABTS cationic free radical scavenging rate equation was as follows:

$$\text{ABTS cationic free radical scavenging rate}\left(\%\right)=\left(1-\frac{A_{\text{sample}}}{B_{\text{control}}}\right)\times 100$$

A_*sample*_ indicates the absorbance of ABTS and sample solutions. A_*control*_ indicates the absorbance of ABTS and methanol solution.

#### 2.7.4 Reducing Power Assay

The reducing power assay was carried out following the procedure described by Lee with modifications (Lee et al. 2021). Mixed 0.2 mL of methanolic extract of yak yogurt, 1 mL of phosphate buffer solution (0.2 mol/L, pH 6.6) and 1 mL of potassium ferricyanide solution (1%, w/w) and reacted at 50°C for 30 min. After cooling, 1 mL of trichloroacetic acid solution (10%, v/v) was added, blended and centrifuged at 3000  × *g* for 10 min. Added 2 mL of supernatant to 0.4 mL of ferric chloride solution (1%, w/w) and stood for 10 min. and measured the absorbance at 700 nm using distilled water as a blank. Absorbance was measured at 700 nm using distilled water as the blank. Reducing power was expressed as the difference in absorbance between the sample solution and the blank control.

### 2.8 Physical Structural Analysis

#### 2.8.1 Texture Analysis

TMS‐Pro texture analyzer (Food Technology Corporation, US) equipped with a 5 kg load cell and a cylindrical extrusion inspection probe (A/BE). The instrument speed was set at 1 mm/s. The test distance, the distance from the surface of the sample, was set at 30 mm. Data was captured at a rate of 200 PPS (packets per second). The textural parameters derived were hardness and consistency.

#### 2.8.2 Microstructural Analysis

Ciron's methodology was followed and appropriately modified (Ciron et al. 2012). The microstructure of yogurt was investigated using confocal laser scanning microscopy (CLSM 900, Zeiss, German) as follows: After dual‐labeling of yogurt with a mixture of Nile Red 0.125%, w/v, in propane‐1,2‐diol and Fast Green FCF (0.1%, w/v, in distilled water), the fat and protein phases were observed separately in the microscope.

### 2.9 Statistical Analysis

This study involved data analysis for all experiments conducted in triplicate, with final results reported as mean and standard deviation (mean ± SEM). Analysis of variance (ANOVA) was performed using SPSS 22.0, employing Duncan's method to assess the significance of differences in the data, with experimental differences analyzed at a 5% significance level. Graphs were plotted using GraphPad Prism 9.

## 3 Results and Discussion

### 3.1 The pH and Acidity of Yak Yogurt Combined With Jujube Syrup During Storage

The acidity in yogurt primarily arises from lactic acid. During the fermentation process, LAB breaks down lactose into lactic acid (Ghorbanzade et al. 2017). While milk can curdle at a certain acidity level, acidity is crucial for yogurt storage; thus, determining the acidity and pH of the sample is a necessary step.

**FIGURE 1:**
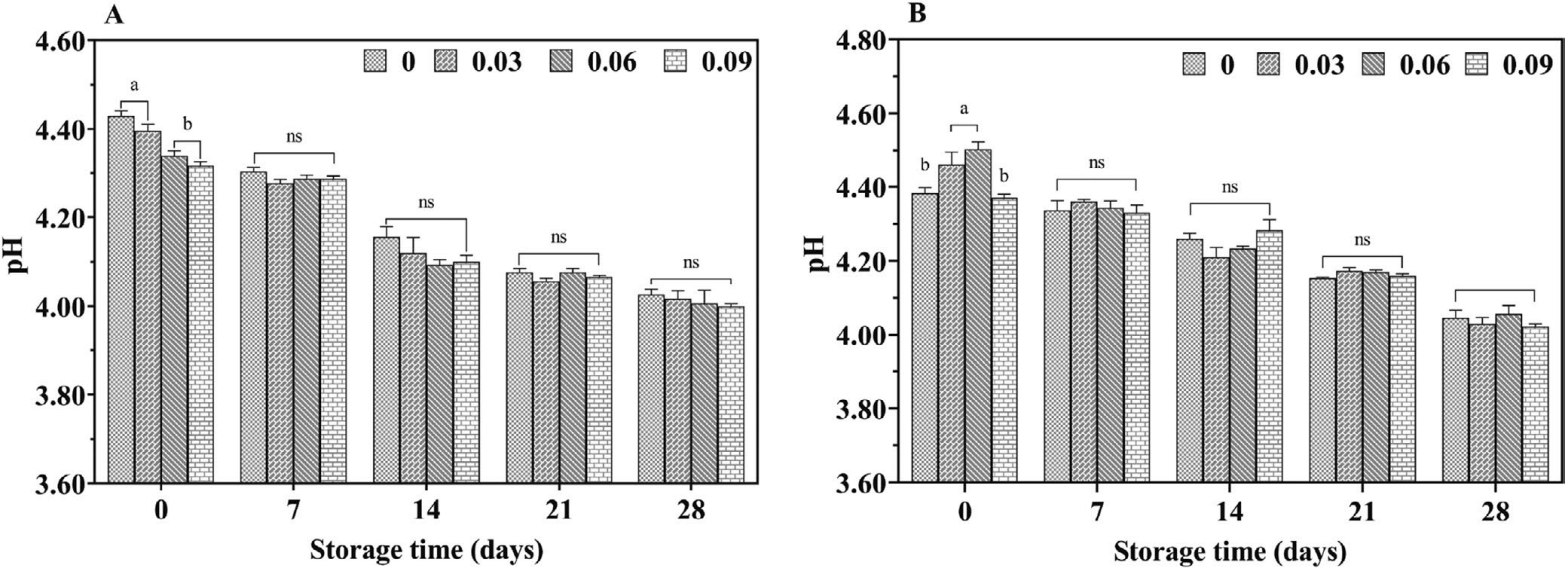
Effect of jujube syrup addition on the pH value of yak yogurt (A) and cow yogurt (B) during storage.

**FIGURE 2:**
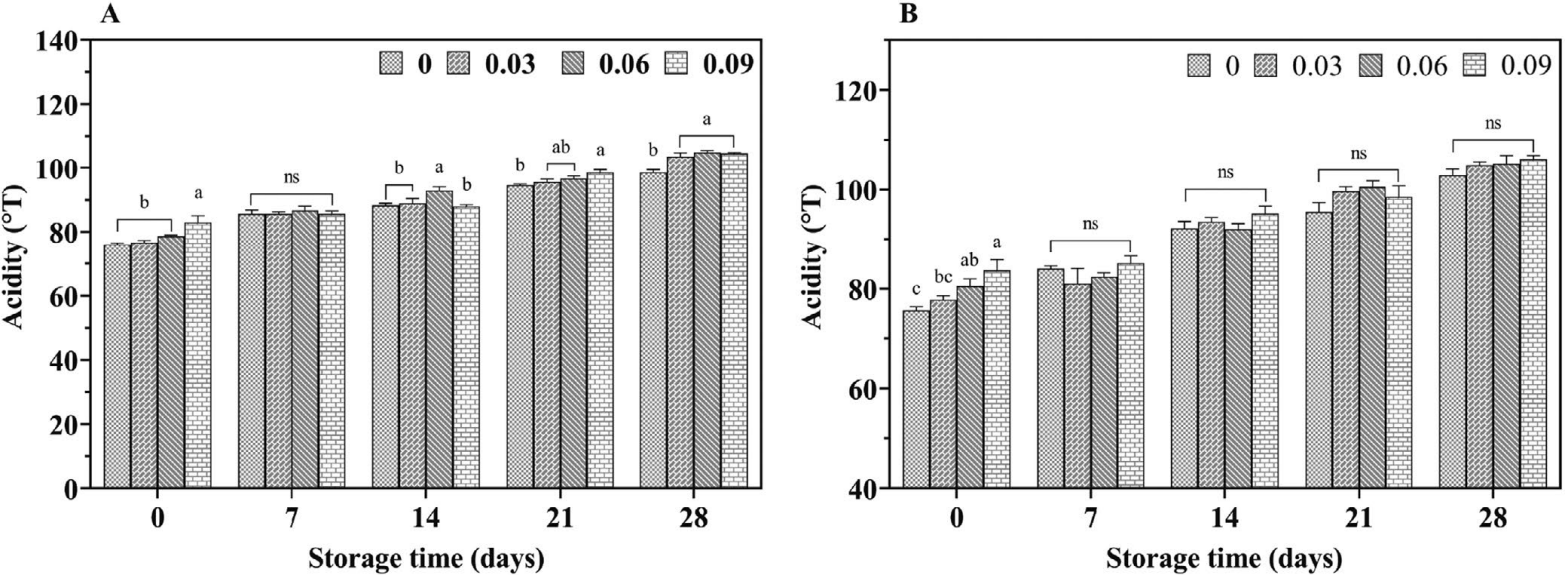
Effect of jujube syrup addition on acidity of yak yogurt (A) and cow yogurt (B) during storage.

In all groups, the pH of yogurt gradually decreased with increasing storage time, while acidity correspondingly increased (Figure 1). When comparing cow yogurt to yak yogurt over the same period, the latter exhibited a lower pH value. The type of yogurt, storage duration and their interactions significantly influence pH and acidity (Figures 1 and 2). Suhartoa et al. studied the changes in pH values of cow yogurt (4.64 ± 0.03 to 4.17 ± 0.21) and goat yogurt (4.50 ± 0.11 to 3.98 ± 0.09) over 15 days of storage (Suharto, Arief, and Taufik 2016). They found that the higher nutrient content of goat milk compared to cow milk resulted in greater acidity due to the fermentation process of LAB. These findings are consistent with the present study, as yak milk, which also has higher fat and protein content than cow milk, achieved greater acidity. Additionally, varying the amount of jujube syrup added did not significantly affect the pH and acidity of yak yogurt (*p* > 0.05), indicating that jujube syrup addition did not significantly influence the acid yield produced by LAB fermentation.

### 3.2 Viable Counts of LAB in Yak Yogurt Combined With Jujube Syrup During Storage

**FIGURE 3:**
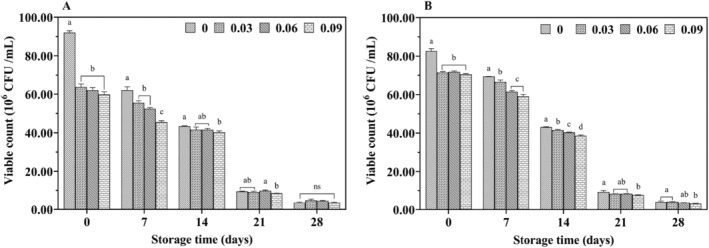
Effect of jujube syrup addition on the viable counts of *LAB* of yak yogurt (A) and cow yogurt (B) during storage.

The live bacterial content of LAB is a key criterion for differentiating product varieties and assessing quality. Figure 3 illustrated the effect of jujube syrup addition on the number of viable LABs during the storage of yak and cow yogurt. The number of viable LAB decreased progressively over the storage period, likely due to the acidity of jujube syrup and the inhibitory effects of polyphenolic compounds on LAB. Additionally, comparing the viable LAB counts in fresh cow milk yogurt and yak milk yogurt revealed that the former had a higher viable LAB count than the latter.

The effect of bioactive components in fruits on the viability of fermenting bacteria can be either positive or negative (Tang et al. 2023), depending on the specific bioactive ingredient and bacterial strain. For example, isoflavones and phytosterols negatively impact probiotic populations (Najgebauer‐Lejko et al. 2021). As described by Tripathi et al. (Tripathi and Giri 2014), the absorption of hydrogen ions by the product's dry matter decreases the pH and increases the concentration of undissociated organic acids, reducing the bactericidal effect on probiotics. Although the number of viable LAB decreased during storage, it remained above 10^6^ CFU/mL, meeting production requirements. This suggested that the addition of jujube syrup did not significantly affect the survival of *B. bulgaricus* and *S. thermophilus*.

### 3.3 Syneresis and Water‐Holding Capacity in Yak Yogurt Combined With Jujube Syrup During Storage

**FIGURE 4:**
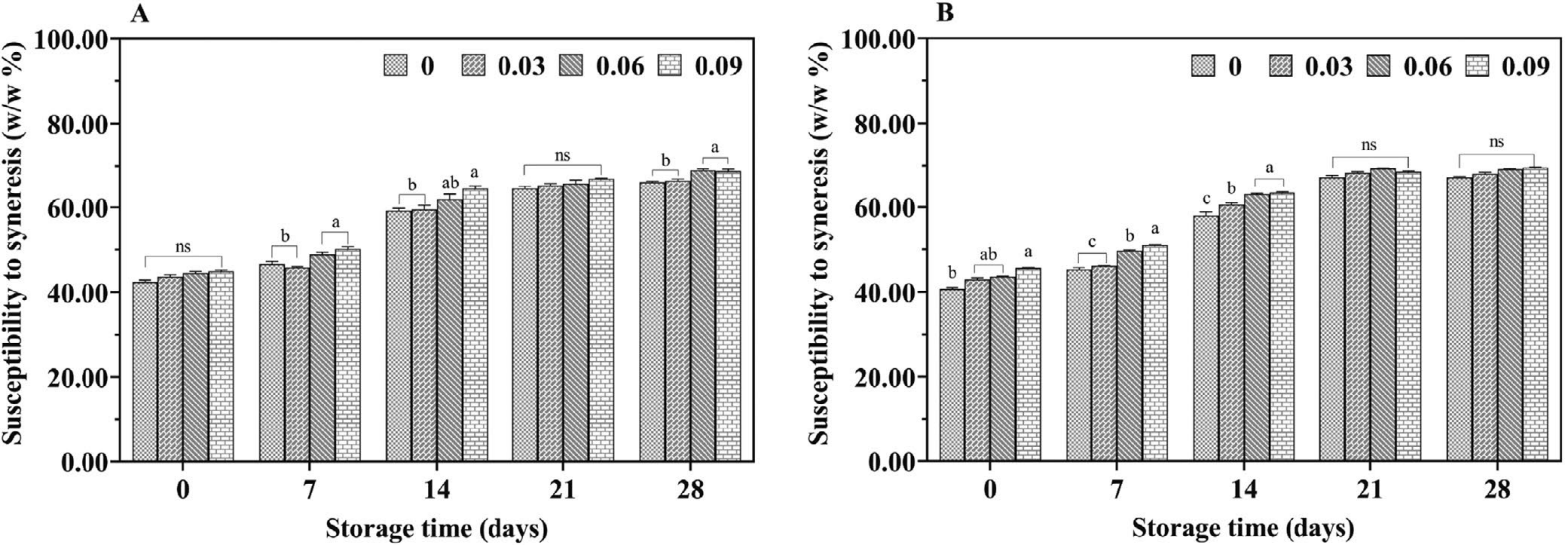
Effect of jujube syrup addition on the syneresis of yak yogurt (A) and cow yogurt (B) during storage.

During storage, the susceptibility to syneresis in jujube syrup yogurt was higher than in cow yogurt without added jujube syrup (Figure 4). It was likely that the addition of jujube syrup promoted dehydration and contraction by disrupting the protein network, reducing the yogurt gel's ability to retain whey. By the end of storage, yogurt containing 9% jujube syrup exhibited the highest level of dehydration and contraction. Throughout the storage period, all samples demonstrated an upward trend in syneresis susceptibility, possibly due to increased acidity, which may have enhanced the dehydrating effect (Sun, Li, and Mei 2021).

**FIGURE 5:**
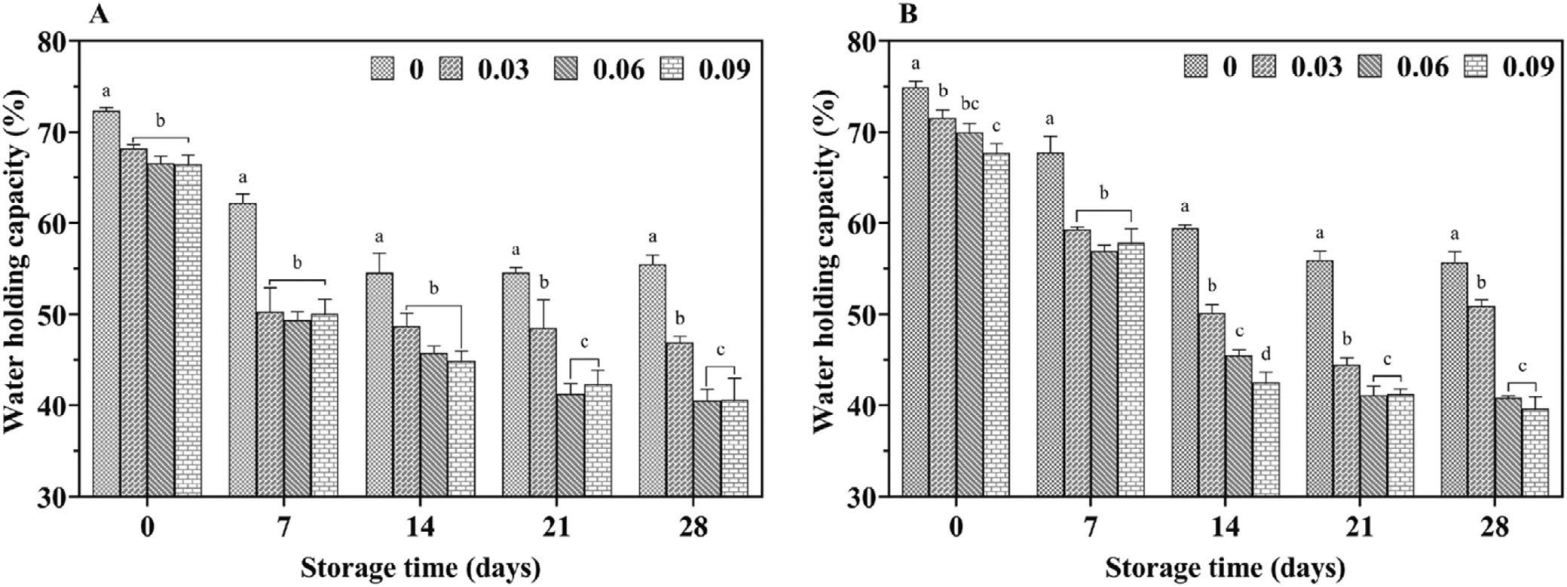
Effect of jujube syrup addition on the water‐holding capacity of yak yogurt (A) and cow yogurt (B) during storage.

The water‐holding capacity of yogurt, which reflects the density of its protein gel network, is closely related to its texture (Ziarno et al. 2023). Weak water‐holding capacity can lead to whey separation, indicating poor yogurt texture and is generally positively correlated with texture quality. The study results showed that both cow yogurt and yak yogurt exhibited similar trends in water‐holding capacity throughout storage (Figure 5). Water‐holding capacity declined gradually over time, with a sharp decrease during the first seven days, followed by a slower decline. Furthermore, the addition of jujube syrup significantly reduced the water‐holding capacity of the yogurt (*p* < 0.05).

The water‐holding capacity of yogurt is primarily influenced by its solids and protein content. Due to the poor consistency of camel milk products, some researchers have used date powder to improve their texture (Jrad et al. 2019). (Farvin et al. 2010) reported that adding fruit juices altered the total solids and protein content of goat's lactic acid milk, affecting its water‐holding capacity (Farvin et al. 2010). In this study, the jujube syrup contained up to 70° Brix of soluble solids, significantly higher than the non‐fat milk solids in yak milk. Therefore, the low water‐holding capacity of yak yogurt with jujube syrup might be attributed to the lower total solids and protein content. Additionally, Moschopoulou et al. found that in yogurt made from sheep, cow, or goat milk, fat globule membrane material enhanced the gel's water‐holding capacity, particularly in full‐fat goat milk yogurt (Moschopoulou et al. 2018).

### 3.4 Sensory Evaluation in Yak Yogurt Combined With Jujube Syrup During Storage

**FIGURE 6:**
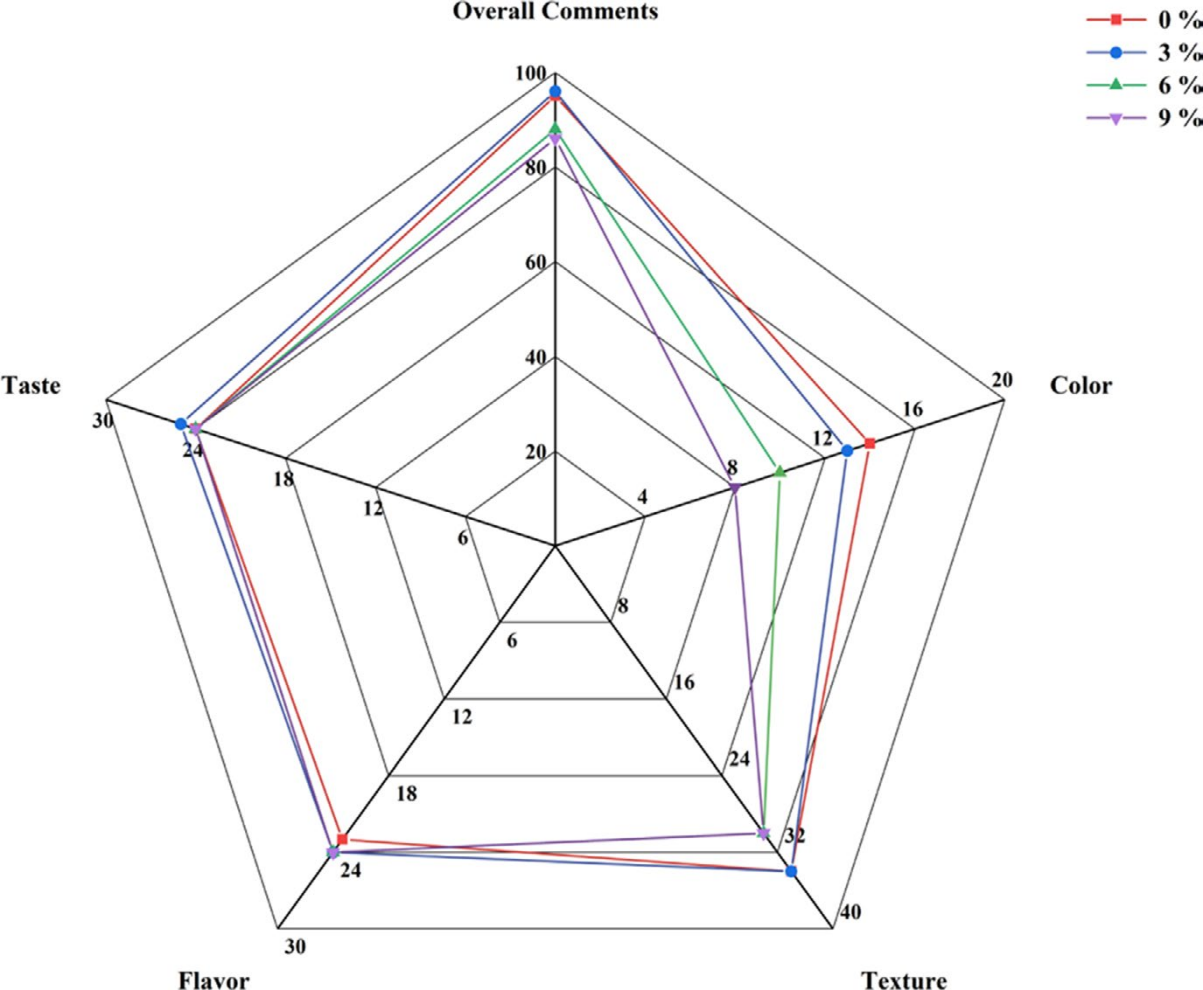
Sensory evaluation of yak yogurt.

The addition of jujube syrup to yak milk improved its sensory quality by masking the undesirable odor and enhancing both flavor and functionality. As shown in Figure 6, the sensory ratings of the yogurt increased initially and then decreased as the volume of jujube syrup increased. Regarding color and flavor, there were no significant differences in sensory ratings between the 3% and 6% jujube syrup additions (*p* > 0.05). However, for flavor, the sensory scores were significantly different from the control group when the jujube syrup content was 3%, 6% or 9% (*p* < 0.05). Similarly, the sensory scores for taste showed significant differences (*p* < 0.05) from the control group at the 6% and 9% addition levels. These results indicate that jujube syrup addition substantially affected the sensory characteristics of yak yogurt.

When 3% jujube syrup was added, the surface of the yogurt remained smooth and delicate, with no whey precipitation and the taste and flavor were optimal, resulting in the highest overall sensory score. Therefore, the 3% addition was the most acceptable in terms of taste and flavor. These combined results indicated that co‐fermentation of yak yogurt with jujube syrup enhanced its flavor, while different concentrations led to varying sensory properties. Appropriate levels of jujube syrup effectively mask the undesirable odor of yak milk, provide a pleasant texture and impart a strong jujube flavor, thereby improving the overall flavor of yak yogurt. Jujube syrup has been shown to enhance the flavor of dairy products. Elamshity et al. demonstrated that jujube syrup improved the properties of flavored milk using VIS–NIR Spectroscopy and ANN Analysis (Elamshity and Alhamdan 2024). Similarly, Shahein et al. found that the addition of jujube syrup as a prebiotic and flavoring agent to probiotic fermented camel milk significantly improved (*p* ≥ 0.05) the sensory scores for flavor, consistency, appearance and overall quality (Shahein et al. 2022). Therefore, jujube syrup, as a natural ingredient, can be successfully applied to yak milk products, improving their quality and facilitating the development of new yak milk products.

### 3.5 Antioxidant Activities in Yak Yogurt With Added Jujube Syrup During Storage

**FIGURE 7:**
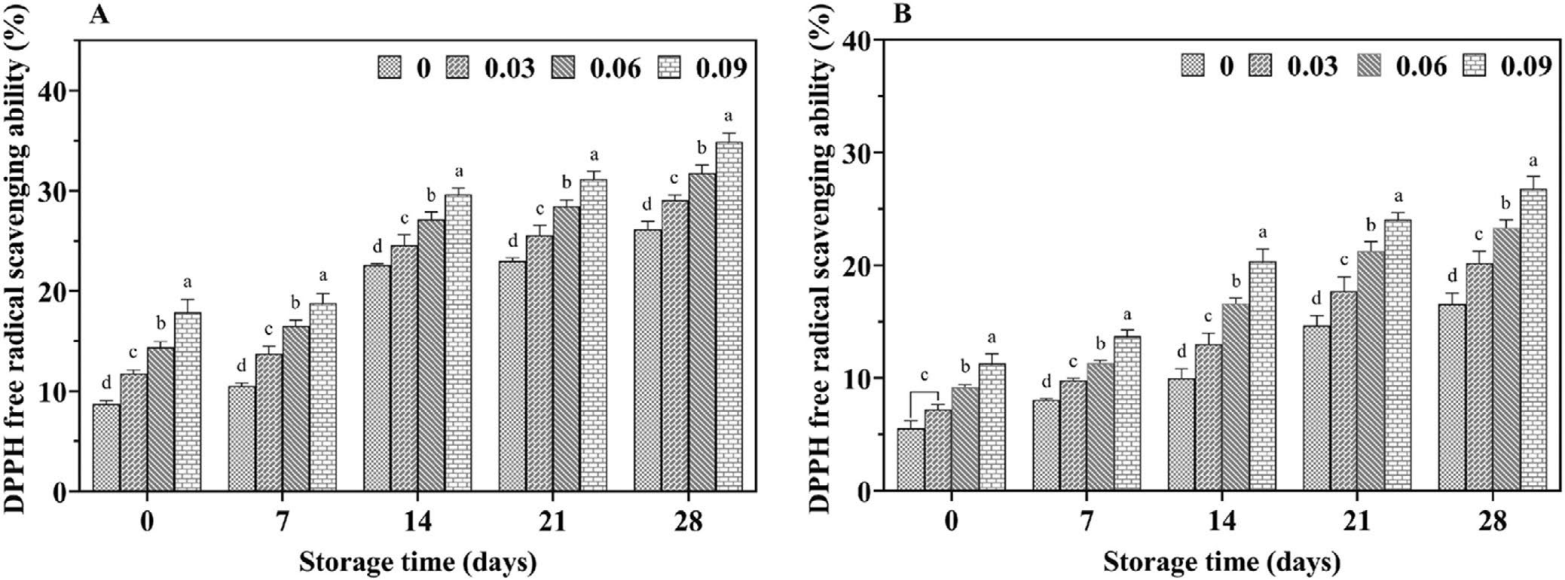
Effect of jujube syrup on DPPH radical scavenging rate of yak yogurt (A) and cow yogurt (B) during storage.

**FIGURE 8:**
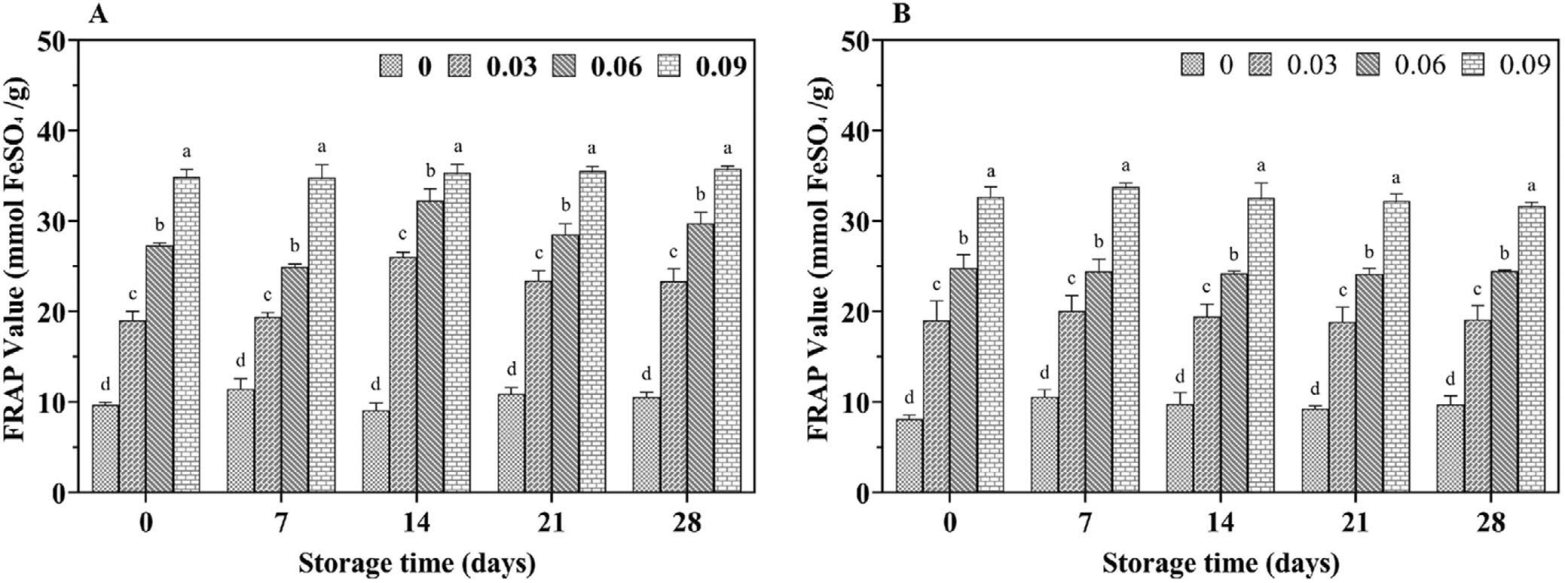
Effect of jujube syrup on the FRAP value of yak yogurt (A) and cow yogurt (B) during storage.

**FIGURE 9:**
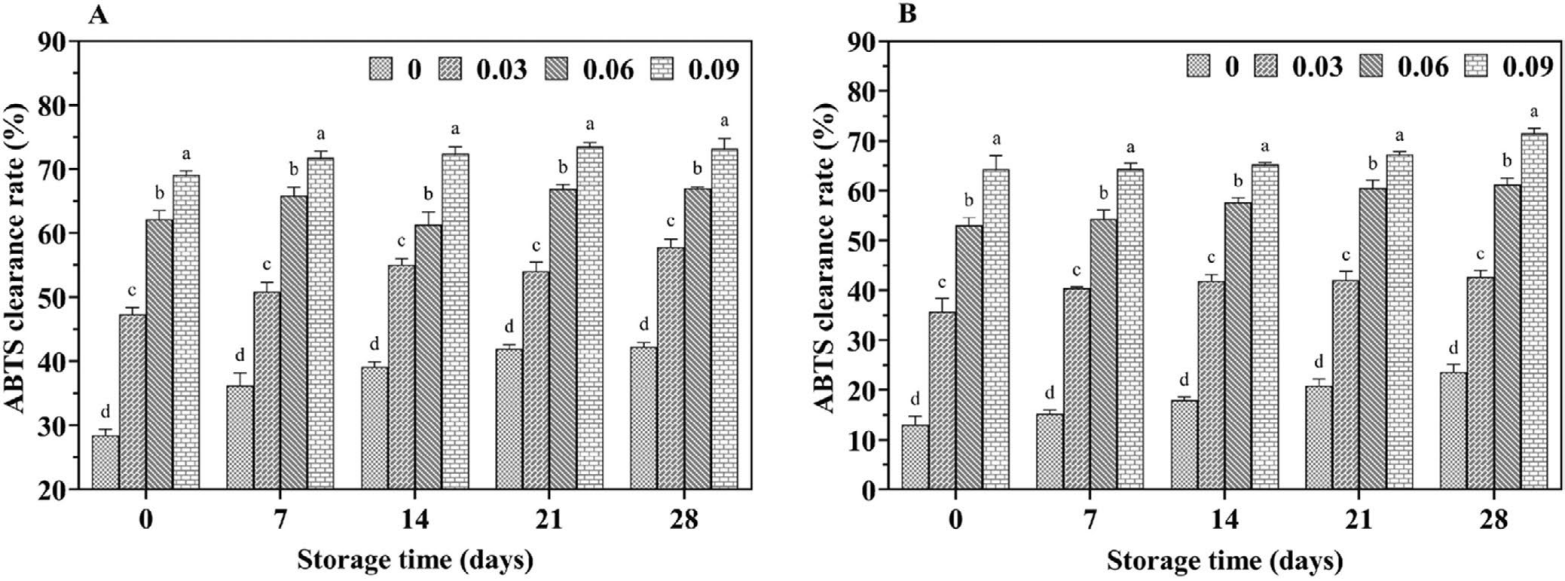
Effect of jujube syrup on the ABTS cationic free radical scavenging capacity of yak yogurt (A) and cow yogurt (B) during storage.

**FIGURE 10:**
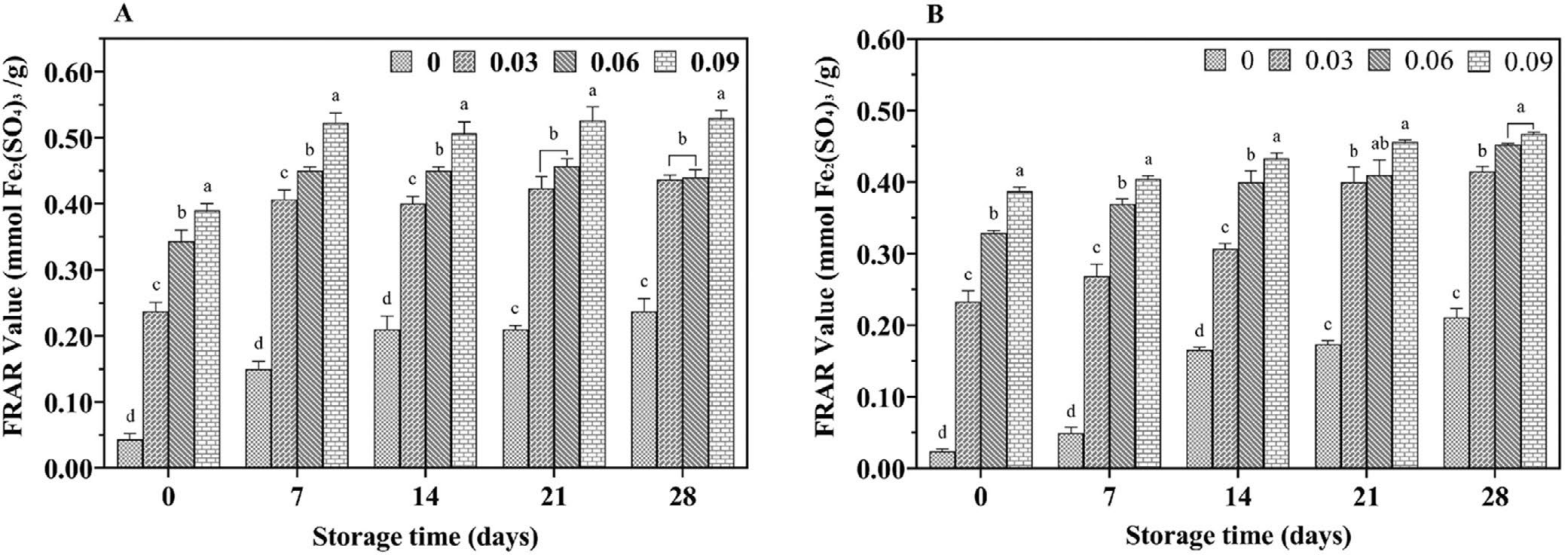
Effect of jujube syrup on the reducing power of yak yogurt (A) and cow yogurt (B) during storage.

With increasing storage time, the DPPH radical scavenging rate (Figure 7) and FRAP values (Figure 8) increased in all yak yogurts, with jujube syrup‐fortified yak yogurts exhibiting higher antioxidant properties than unfortified ones. Additionally, a significant positive correlation (*p* < 0.05) was observed between the scavenging rate and FRAP values with increasing levels of jujube syrup. The scavenging capacity of ABTS cationic radicals and the reducing power were shown in Figures 9 and 10, respectively. Both the ability to scavenge ABTS cationic radicals and the reducing power increased with the addition of jujube syrup, though these properties remained relatively unchanged with extended storage periods. Moreover, the ability to scavenge DPPH and ABTS cationic radicals were higher in yak yogurt than in cow yogurt. The findings indicated that higher levels of jujube syrup significantly enhanced the scavenging of DPPH radicals, ABTS cationic radicals, FRAP values and ferric ion reducing power.

The higher antioxidant activity of yak yogurt compared to cow milk yogurt (Figures 7, 8, 9, 10) could be attributed to the higher levels of amino acids and small peptides in yak milk. The antioxidant activity of yogurt is primarily due to the amino acids and small peptides produced during fermentation (Farvin et al. 2010). The addition of jujube syrup further enhanced the antioxidant activity of the yogurt, likely due to the increased phenolic compound content. Phenolic compounds, which act as natural antioxidants by absorbing free radicals, were hypothesized to improve the antioxidant capacity of the yogurt. Similar improvements in antioxidant activity were observed when plant additives such as dill, green tea extract and barley were added to yogurt (Li, Duan et al. 2023; Muniandy, Shori, and Baba 2016; Tizghadam et al. 2021). Amirdivani reported that plant polyphenols significantly boost yogurt's antioxidant activity (Amirdivani and Baba 2011). And jujube, rich in polyphenols, further enhances the antioxidant capacity of yogurt (Granato et al. 2018). Jrad et al. also demonstrated that jujube powder improved the antioxidant properties of camel yogurt (Jrad et al. 2019). In addition to polyphenols, the vitamin C content in jujube syrup contributes to its antioxidant effects. Gad et al. found that adding jujube syrup to functional yogurt significantly increased its vitamin C content (Gad, Kholif, and Sayed 2010). Similarly, Shahein reported that fermented camel milk with 8% date syrup exhibited total phenolic and antioxidant activities that were 40 and 5 times higher, respectively, than fermented camel milk without the syrup (Shahein et al. 2022). The vitamin C content was also significantly higher in the date syrup‐enriched camel milk. Therefore, yak yogurt containing polyphenolic compounds exhibited strong antioxidant activity.

### 3.6 Structural Analysis in Yak Yogurt With Added Jujube Syrupy During Storage

**FIGURE 11:**
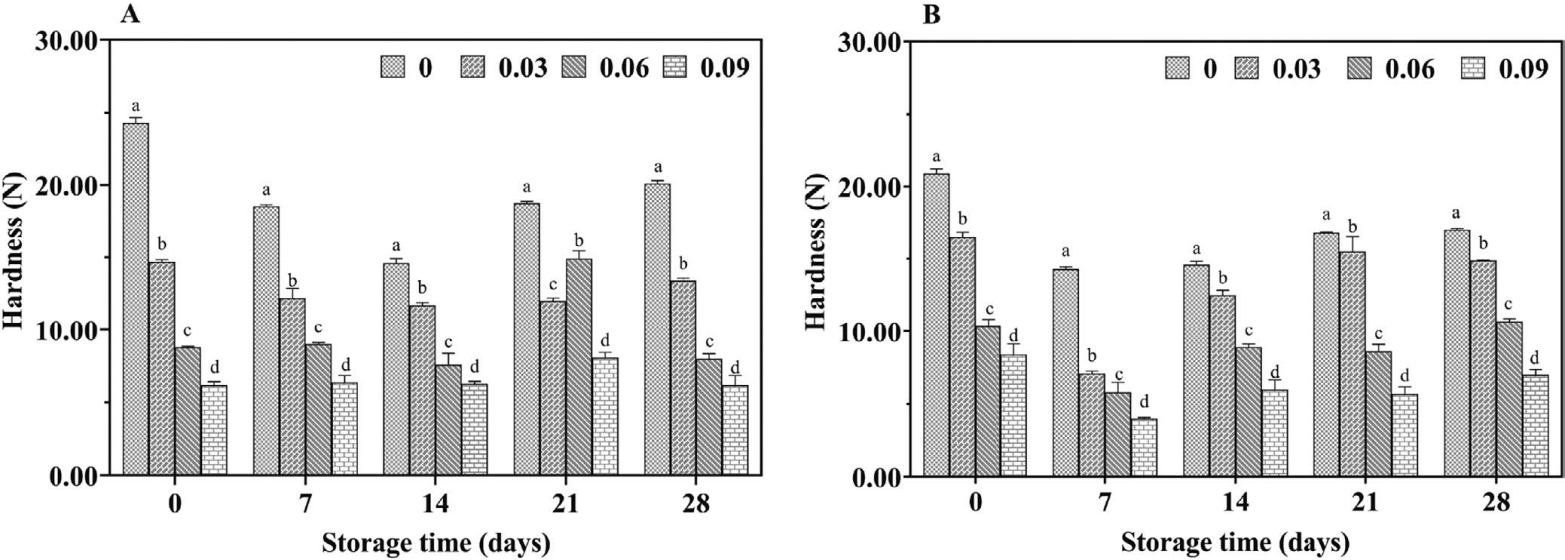
Effect of jujube syrup on the hardness of yak yogurt (A) and cow yogurt (B) during storage.

**FIGURE 12:**
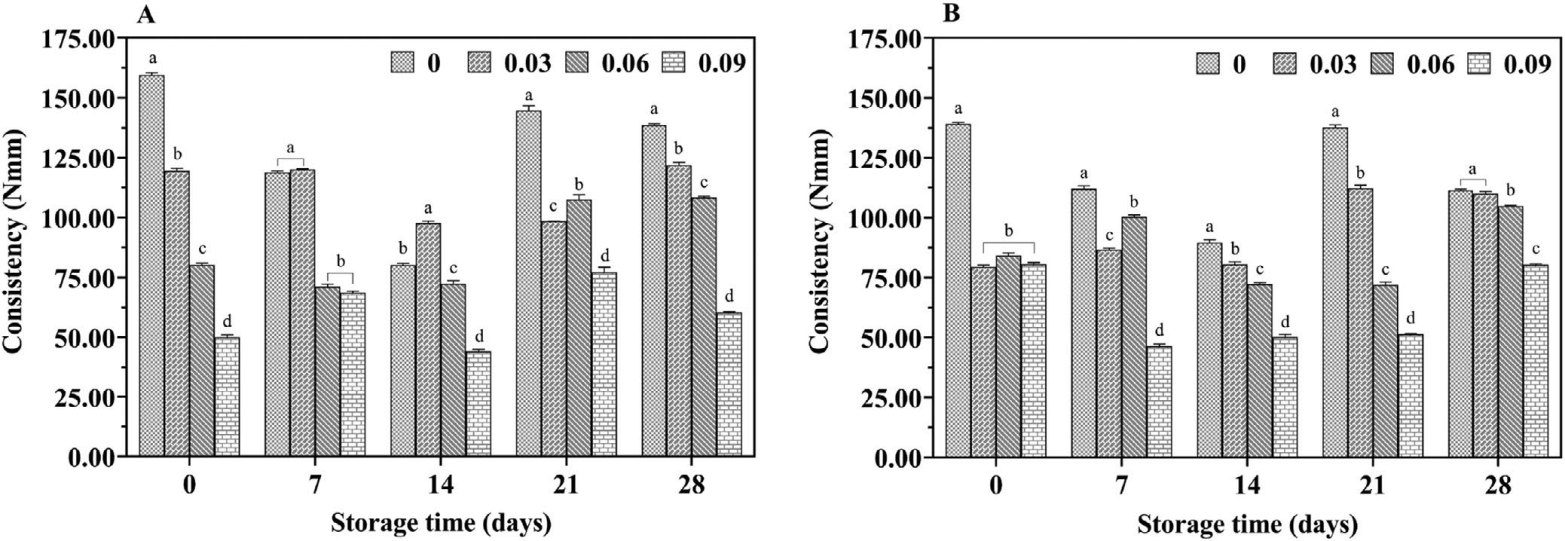
Effect of jujube syrup on the consistency of yak yogurt (A) and cow yogurt (B) during storage.

Texture characterization is a key parameter in evaluating yogurt quality. The hardness of yogurt directly indicates its gel formation, with greater hardness correlating to better curdling. Consistency is measured by the stickiness of the yogurt to the testing probe. Figures 11 and 12 illustrates the effect of jujube syrup addition on the textural properties of yak yogurt during storage.

The hardness trends of cow yogurt and yak yogurt were found to be similar. The hardness of yak milk yogurt gradually decreased with increasing jujube syrup concentrations and this decrease was statistically significant (*p* < 0.05) (Figure 11). Previous studies suggested that the hardness of fermented milk was closely linked to the culture composition (Oliveira et al. 2001). Additionally, as the jujube syrup concentration increased, the consistency of all yak milk yogurts also decreased, with significant differences between the different concentrations (*p* < 0.05) (Figure 12). This reduction in consistency could be attributed to the polysaccharides in the jujube syrup. Notably, yogurt with 9% jujube syrup exhibited significantly lower consistency than yogurt with 0% syrup, likely due to the higher total soluble solids content of the syrup, which increased osmotic pressure, inhibited the growth of LAB and reduced yogurt gel formation. These results aligned with Costa's findings (Costa et al. 2015).

**FIGURE 13:**
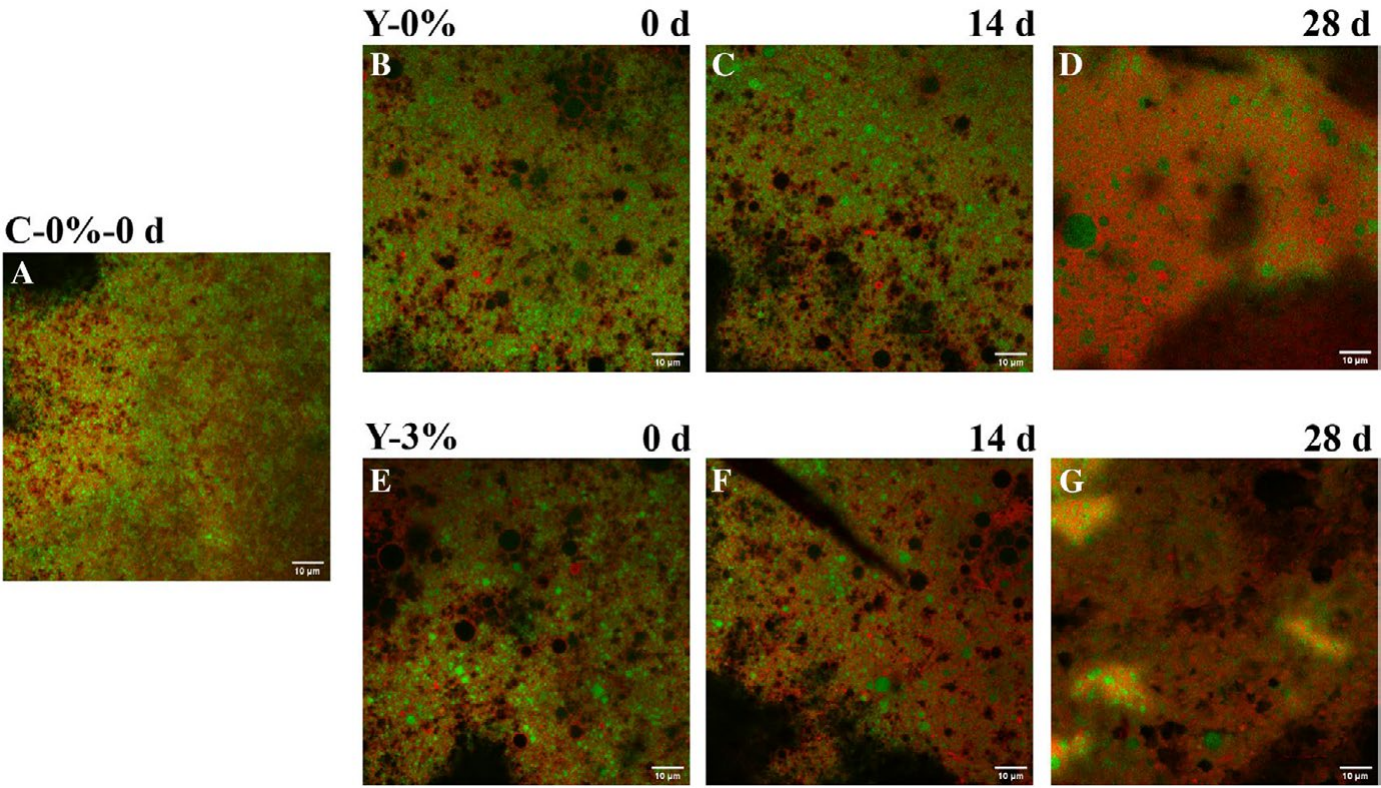
Microstructure of cow yogurt, yak yogurt and 3% jujube syrup yak yogurt observed by CLSM during storage. (A, cow yogurt on day 0; B–D, yak yogurt on day 0, day 14 and day 28; E–G, 3% jujube syrup yak yogurt on day 0, day 14 and day 28).

CLSM revealed that the addition of jujube syrup and the storage period affected the microstructure of yak yogurt (Figure 13). Nile Red‐stained fat appeared red, Fast Green FCF‐stained protein appeared green and the black areas represented whey pores. The merging of the red and green channels created a superimposed image, allowing clear differentiation of the fat phase. The size and distribution of fat globules differed between cow and yak yogurt. However, no significant difference in fat globule size was observed between the two in this study. Gupta et al. found that yogurt with high‐fat coconut content had larger fat globules (~10 μm in diameter), while drinkable and berry yogurts had smaller fat globules more integrated into the protein network, likely due to high‐shear homogenization (Gupta et al. 2022). Homogenization reduces the size of milk fat globules and enhances interaction with milk proteins, contributing to yogurt consistency. In this study, fat globules in yak yogurt were more abundant than in cow yogurt (Figure 13A,B), consistent with yak milk's higher fat content compared to Holstein milk (Agyare and Liang 2021). Larger whey pores (black areas) were visible in all samples, but they were more prevalent in yak yogurt (b–g). The observation suggested that yak milk yogurt was more susceptible to whey separation. Notably, 3% jujube syrup yak yogurt exhibited a higher number of whey pores. It was reported that larger pores were present in the protein network with lower density (Nguyen et al. 2014). The present study suggests that jujube syrup affected hydrophobic interactions within casein particles, leading to a microstructure with larger whey pores.

## 4 Conclusion

The co‐fermentation of yak yogurt with jujube syrup led to significant changes in its physicochemical properties, sensory characteristics and antioxidant activity during storage. As the amount of jujube syrup increased, the antioxidant capacity and free radical scavenging ability of yak yogurt also increased. Jujube syrup influenced syneresis, texture and the microstructure of the yak yogurt, with the optimal sensory score and overall acceptability in terms of water‐holding capacity and texture being achieved at 3% jujube syrup. Additionally, throughout the storage period, the viable bacterial count in all yak yogurt samples remained above the required threshold (> 10^6^ CFU/mL), ensuring potential health benefits for consumers. Therefore, a 3% addition of jujube syrup is recommended, as it imparts a smooth, delicate texture, a delicious taste and a rich jujube flavor to the yogurt. Ultimately, the study successfully demonstrated that incorporating jujube syrup into yak milk products can enhance both the nutritional and sensory quality while increasing market value due to the functional benefits of red jujubes and yak milk.

## Author Contributions

**Xiaolin Liang:** data curation (lead), writing – original draft (equal). **Bo Ding:** visualization (lead), writing – review and editing (equal). **Songxuan Li:** investigation (lead). **Hao Zhang:** funding acquisition (equal), resources (lead). **Jialin Bai:** supervision (equal). **Jutian Yang:** supervision (equal). **Dandan Gao:** methodology (equal). **Jiajia Song:** writing – review and editing (equal). **Hongna Liu:** conceptualization (equal), methodology (equal).

## Ethics Statement

The authors have nothing to report.

## Consent

Written informed consent was obtained from all study participants.

## Conflicts of Interest

The authors declare no conflicts of interest.

## Data Availability

The data that support the findings of this study are available from the corresponding author (Dr. Liu Hongna), upon reasonable request.
